# Bioinorganic Chemistry of Micronutrients Related to Alzheimer’s and Parkinson’s Diseases

**DOI:** 10.3390/molecules28145467

**Published:** 2023-07-17

**Authors:** Arian Kola, Federico Nencioni, Daniela Valensin

**Affiliations:** Department of Biotechnology, Chemistry and Pharmacy, University of Siena, Via Aldo Moro 2, 53100 Siena, Italy; arian.kola@unisi.it (A.K.); federico.nencioni2@unisi.it (F.N.)

**Keywords:** bioinorganic chemistry, metal ions, vitamins, vitamin-metal complexes, neurodegeneration, Alzheimer’s disease, Parkinson’s disease, copper, iron, metal dyshomeostasis

## Abstract

Metal ions are fundamental to guarantee the regular physiological activity of the human organism. Similarly, vitamins play a key role in many biological functions of the metabolism, among which are coenzymes, redox mediators, and antioxidants. Due to their importance in the human organism, both metals and vitamins have been extensively studied for their involvement in neurodegenerative diseases (NDs). However, the full potential of the interaction between vitamins and metal ions has not been fully explored by researchers yet, and further investigation on this topic is needed. The aim of this review is to provide an overview of the scientific literature on the implications of vitamins and selected metal ions in two of the most common neurodegenerative diseases, Alzheimer’s and Parkinson’s disease. Furthermore, vitamin–metal ion interactions are discussed in detail focusing on their bioinorganic chemistry, with the perspective of arousing more interest in this fascinating bioinorganic field.

## 1. Introduction

The human organism is primarily made up of water, fat, proteins, and minerals [1]. Metals are present in small quantities in the body. For example, an adult male body contains 3–4 g of iron [2]. Nevertheless, metals are necessary for the preservation of physiological functions of the organism. They are involved in several biological processes such as electron transfer, oxygen transport, the maintenance of osmotic pressure, and the regulation of DNA transcription [3]. Metals such as iron, cobalt, selenium, copper, zinc, and manganese are essential for human life and are usually required in trace amounts. On the other hand, aluminum, mercury, arsenic, and others are considered non-essential metals since they possess no biological function [4,5]. The importance of metals in the human organism is so fundamental that several pathologies, among which are neurodegenerative diseases (NDs), are related to a common phenomenon known as metal dyshomeostasis [6]. The scientific literature offers a large number of papers addressing the implications of metals in NDs [7,8,9].

Metal ions have been extensively studied for their interactions with important biomolecules such as amino acids [10,11], peptides [12,13], and proteins [14,15], which are involved in structural functions, cell signaling, cell expression, and hormone synthesis, to name a few [16,17]. Proteins often need to interact with metal ions to carry out their function [18].

As for metal ions, vitamins play a key and fundamental role in the healthy state of the human organism. This class of compounds, vital for existence, are essential micronutrients [19], although niacin [20] and vitamin D_3_ [21] can be synthesized by the organism. Vitamins are involved in many important biological functions in the human metabolism such as (i) coenzymes, (ii) redox mediators, (iii) antioxidants, (iv) hormones, and (v) regulators of gene transcription [22].

As with metals, the role of vitamins in neurodegeneration has been a hot research topic during recent decades [9,23]. However, the number of studies on the interaction between metal ions and vitamins is not comparable to the research concerning metal–amino acid, metal–peptide, and metal–protein interactions. This field is just at the beginning but possesses all the features to offer new insight to improve the knowledge about the interactions between vitamins and metal ions in biological systems.

In view of the above, the aim of this review was to contribute to this topic by giving an overview of the literature about the role of vitamins and selected metal ions in the two most common neurodegenerative diseases: Alzheimer’s and Parkinson’s diseases. In addition, the chemical interaction between vitamins and metal ions was addressed to evaluate the role of these associations and their impact on healthy homeostatic equilibria.

## 2. Vitamins in Parkinson’s and Alzheimer’s Diseases

Nowadays neurodegenerative diseases are recognized as the most severe brain disorders characterized by a loss of neuron structure and function in specific brain areas, which ultimately leads to neuronal death. Neurodegeneration greatly impacts ordinary life due to the gradual cognitive decline primarily associated with speech, motor, and memory faculties. The most common NDs are Alzheimer’s disease (AD) and Parkinson’s disease (PD), affecting millions of people worldwide [24,25]. Both diseases are strongly correlated with aging, considered the most impacting risk factor for the development of neurodegeneration [26,27]. Moreover, with the increasing life expectancy in the elderly population, the prevalence of both AD and PD has been growing year after year [28]. The major critical hallmarks of AD and PD are protein aggregation, synaptic and neuronal network dysfunction, abnormal protein homeostasis, cytoskeletal defects, mitochondrial dysfunction, altered energy metabolism, DNA and RNA defects, inflammation, and neuronal death [25,29,30].

**Figure 1:**
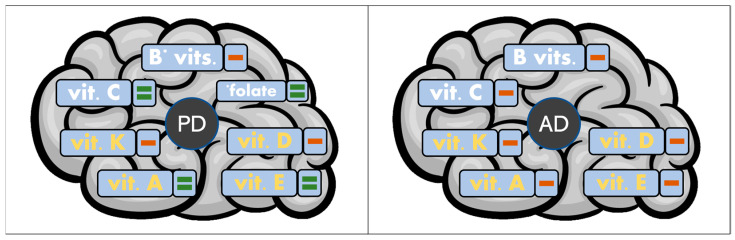
Schematic representation of vitamin impairment in PD and AD. Water- and fat-soluble vitamins are highlighted in white and yellow, respectively. “=” indicates no significant difference between vitamin levels of sick subjects and healthy controls, while “−” refers to lower vitamin levels in patients with PD or AD.

In this scenario, many biomolecules are extensively impaired in both AD and PD. Among them, vitamins are known to be implicated in several cellular processes associated with NDs by acting as antioxidant and anti-inflammatory compounds [23,31,32]. Extensive research has been performed in this field, underlining the role of vitamins on healthy and neurodegenerative brains. Most of the investigations focused on understanding abnormal vitamin levels associated with AD and PD and evaluating the protective effects of vitamin administration. The most recent results are hereafter discussed and schematized in Table 1 and Table 2 and Figure 1.

### 2.1. Parkinson’s Disease

Several clinical and case-control studies have correlated PD with vitamin B deficiency, also associated with an increased risk of the disease’s onset (Table 1) [33,34,35,36,37,38,39,40]. Low serum levels of vitamins B_6_ and B_12_ have been detected in PD cases characterized by polyneuropathy, epilepsy, and cognitive impairments [33]. Concentrations of pantothenic acid (vitamin B_5_) were dramatically changed in PD post-mortem brains, showing an approximately 40% reduction in the substantia nigra and the cerebellum regions [41]. Moreover, the implication of vitamins B_1_, B_2_, B_6_, B_9_, and B_12_ in PD also emerged from case-control and population-based cohort studies, showing that (i) low levels of vitamin B_6_ correlate with higher PD risk [36,37]; (ii) vitamin B_1_ and vitamin B_9_ are involved in olfactory dysfunctions in the early phase of PD [34], and (iii) mild cognitive impairment (MCI) in PD is associated with plasma levels of thiamine (vitamin B_1_) and thiamine-monophosphate, which are differently influenced according to sex [35]. Finally, recent studies focusing on the comprehension of neurotoxic pathways induced by biotin (vitamin B_7_) deficiency show that the absence of said vitamin causes the extensive formation of vacuoles in the brain, locomotor deficits, reduced levels of biotin-dependent carboxylases, elongated mitochondrial conformation, and increased superoxide levels [38].

In addition to the low levels of B vitamins associated with PD, a plethora of studies has also supported the neuroprotective activity of B vitamins against PD and other neurodegenerative disorders. In most cases, B vitamin administration led to an improvement in PD symptoms as observed by population-based cohort studies and clinical and animal in vivo investigations (Table 1) [36,42,43,44,45]. In particular, long-term intramuscular administration of thiamin led to the significant and stable amelioration of motor and non-motor capacity in PD patients [42]. Similarly, oral intake of riboflavin (vitamin B_2_) in 19 PD cases resulted in improvements in motor functions [43]. Analogous results were obtained using *Drosophila* PD [44] and *C. Elegans* models [45] whose motor abilities were enhanced upon administration of vitamin B_3_ and B_12_, respectively.

In health status, B vitamins are involved in specific metabolic pathways, thus playing an essential role in regulating mitochondrial enzymes. The lower levels (Table 1 and Table 2) observed in both PD and AD (vide infra) correlate well with the compromised mitochondrial activity usually observed in neurodegeneration. For example, the reduction of α-ketoglutarate dehydrogenase complex (αKGDC) activity is implicated in several NDs [46,47]. αKGDC, requiring vitamin B_1_ as a cofactor, is involved in the Krebs cycle, regulating multiple cellular processes such as reactive oxygen species (ROS) generation and inhibition [48,49]. Vitamin B_6_ acts as a cofactor in a huge number of cellular biochemical reactions [50,51]. It is involved in the synthesis of neurotransmitters including dopamine whose levels are significantly reduced in PD brains. Vitamin B_6_ is also required for the conversion of homocysteine (Hcy) to cysteine and, together with vitamin B_12_ and folate (vitamin B_9_), plays a key role in maintaining physiological plasma levels of Hcy (10–20 mol/L) [52]. Interestingly, higher Hcy concentrations in the blood are considered a major risk factor for AD, PD, and vascular dementia [53,54,55].

The protective effects of vitamin supplementation on mitochondrial dysfunctions, oxidative stress, inflammation, and neurotoxicity are also supported by in vitro studies [38,44,45,56]. In particular, the anti-inflammatory activity exhibited by niacin (vitamin B_3_) was correlated with the inhibition of inflammatory cytokines and its G-protein-coupled receptor GPR109A [56], usually up-regulated in PD white patients [57]. On the other hand, research studies on the SH-SY5Y PD model have indicated that the antioxidant activity of vitamin B_12_ is dependent on the regulation of p-JNK and PGC-1α levels, which are usually altered in oxidative stress conditions [45].

Over the last few years, scientific research on vitamin D and its relationship with PD has exploded in terms of contributions. On this issue, a large number of reviews have been published in the last five years (see, for example, [58,59,60,61,62]). Several studies have shown low vitamin D levels in PD patients along with a connection between high vitamin D concentration and reduced risk of PD [63,64]. In a mouse model of PD, subcutaneous administration of vitamin D_3_ led to the attenuation of behavioral deficits induced by 6-hydroxydopamine [65]. The role played by vitamin D in brain development is well known, such that a daily intake of 600 UL of vitamin D is recommended for pregnant women [66]. Moreover, vitamin D supports those neuronal and glial functions necessary to preserve neurological development and protect the adult brain [67]. In fact, vitamin D is essential for the neuronal survival of hippocampal and cortical neurons by regulating the release of nerve growth factor [68] and it is involved in dopaminergic neurogenesis and differentiation [69].

Currently, the knowledge of the relationship between PD and vitamin K is quite limited, although recent findings have attracted the scientific community to the possible therapeutic use of vitamin K in NDs [70]. Vitamin K is implicated in neuronal development and survival by modulating sphingolipid metabolism whose alteration leads to neuroinflammation and neurodegeneration [71]. A case-control study reported lower serum levels of vitamin K_2_ in PD patients when compared to controls [72]. In a *Drosophila* PD model, feeding with vitamin K_2_ significantly ameliorated the survival rate of the flies. In addition, vitamin K_2_ rescued mitochondrial dysfunction by acting as an electron transporter in mitochondria and maintaining efficient adenosine triphosphate (ATP) production [73]. The neuroprotective effects exhibited by vitamin K are further supported by its ability to (i) regulate the mitochondrial membrane potential and (ii) inhibit dopamine neuron damage caused by 6-hydroxydopamine [74] and activate the GAS6 protein, fundamental for cellular growth, survival, and death [75]. Finally, in vitro experiments have shown that vitamin K can reduce α-synuclein (αSyn) fibrillization at a substoichiometric concentration as shown by ThT fluorescence and AFM experiments [76]. At the same time, NMR structural investigations provided evidence of vitamin K interactions with αSyn. The most perturbed chemical regions are Gly31, Lys32, and 1,4-naphthoquinones for α-synuclein and vitamins K, respectively [76]. Since the N-terminal repeat region of αSyn is critical for fibril formation and morphology, the binding of vitamin K to this region might lead to structural rearrangements capable of interfering with protein fibrilization.

Similar to vitamin K, vitamin A is able to reduce αSyn fibrilization as well [77]. However, in contrast to vitamin K, no specific and selective protein-vitamin association has been found for the investigated retinol derivatives so far. Despite this, in vivo animal studies have indicated that the intracerebroventricular treatment with retinoic acid had a protective effect against the degeneration of dopaminergic neurons [78]. On the other hand, oral administration of retinol did not protect 6-hydroxydopamine-induced dopaminergic denervation in Wistar rats [79].

Vitamins A, C, and E are powerful antioxidant compounds able to scavenge peroxyl radicals. Among them, vitamin E is the most effective one, being more active than glutathione (GSH) and ß-carotene [80,81]. Both vitamins C and E are normally highly concentrated in the brain where they exert their radical scavenging activity. In fact, vitamin C feeding was found to promote catalase activity and reduce protein oxidation and H_2_O_2_ production in a *Drosophila* model of PD [82]. Moreover, in a rotenone-induced model of PD, intramuscular vitamin E treatment showed neuroprotective effects by (i) improving motor functions, (ii) reducing lipid peroxidation, and (iii) ameliorating GSH and superoxide dismutase (SOD) levels [83].

By considering the antioxidant role played by vitamins A, C, and E, reduced levels of these vitamins are expected to be associated with PD, characterized by elevated oxidative stress conditions. However, no clear results or consensus are available so far. A clinical study conducted with 44 PD patients has indicated no difference in vitamins C and E levels between healthy and PD cases [84]. On the other hand, reduced levels of lymphocyte vitamin C were observed in patients with severe PD [85]. The same uncertainty is found between two recent studies reporting the results based on a population-based cohort [86] and a meta-analysis [87]. The former identified no association between dietary intake of A, C, and E vitamins and the risk of developing PD. The latter found protective effects of vitamin E supplementation. However, the same authors do not exclude that such results could be due to other contributions, such as lifestyle and behavioral factors that were not taken into account in these studies.

**Table 1:** Summary of the studies and the effects (↑ increase; ↓ decrease; ↔ no change) of vitamins in PD.

Vitamin	Type of Study	Intervention	Results	Ref.
B_1_	Case-control	Plasma levels	↓ B_1_	[35]
B_6_, B_9_, B_12_	Rotterdam	Dietary intake	↑ B_9_, B_12_ ↔ PD risk; ↑ B_6_ ↓ PD risk	[36]
B_2_, B_6_, B_9_, B_12_	Case-control	Dietary intake	↑ B_2_, B_9_, B_12_ ↔ PD risk; ↓ B_6_ ↑ PD risk	[37]
B_7_	In vivo	*Drosophila*	↓ B_7_	[38]
		Rodents	↓ Biotin carboxylase	
	In vitro	Neurons	↓ B_7_ ↑ Mitochondrial stress	
B_12_	Clinical	Serum levels	↑ B_12_ ↓ PD risk	[40]
B_5_	Ex vivo	Human PD brains	↓ B_5_	[41]
B_1_	Clinical	Intramuscular administration	↑ B_1_ ↑ Motor and non-motor functions	[42]
B_2_	Clinical	Oral administration	↑ B_2_ ↑ Motor functions	[43]
B_3_	In vivo	*Drosophila*	↑ B_3_ ↑ Motor functions	[44]
	In vitro	SK-N-MC neurons	↑ B_3_ ↓ Cytotoxicity	
B_12_	In vivo	*C. elegans*	↑ B_12_ ↓ ROS ↑ Motor functions	[45]
		Rodents	↑ B_12_ ↑ Motor functions;	
	In vitro	SH-SY5Y neurons	↑ B_12_ ↓ Ox. stress ↓ Apoptosis	
B_3_	In vitro	RAW264.7 cells	↑ B_3_ ↓ Neuroinflammation	[56]
D	Cross-sectional	Serum levels	↓ D	[64]
D_3_	In vivo	Rodents	↑ D_3_ ↑ Motor and non-motor functions ↓ Ox. stress	[65]
K_2_	Case-control	Serum levels	↓ K_2_	[72]
K_2_	In vivo	*Drosophila*	↑ K_2_ ↑ Survival rate	[73]
	In vitro	Mitochondria	↑ K_2_ ↑ ATP	
K	In vitro	αSyn	↑ K ↓ Fibrillization	[76]
A	In vivo	Rodents	↑ A ↑Neuroprotection	[78]
	In vitro	E14–15 neurons	↑ A ↑ Viability	
A	In vivo	Rodents	↑ A ↔ Neuroprotection	[79]
C, E	In vivo	*Drosophila*	↑ C, E ↓ Ox. stress	[82]
E	In vivo	Rodents	↑ E ↑ Neuroprotection ↑ Motor functions	[83]
A, C, E	Clinical	Plasma levels	↔ A, C, E	[84]
C	Clinical	Lymphocyte levels	↓ C	[85]
A, C, E	Prospective cohort	Dietary intake	↑ A, C, E ↔ PD risk	[86]

### 2.2. Alzheimer’s Disease

Similar to PD, B vitamin deficiency was observed in AD cases as well (Table 2). Clinical studies on AD patients have indicated that lower concentrations of vitamins B_9_ and B_12_ correlate with high serum Hcy levels [88,89]. As previously mentioned, hyperhomocysteinemia is commonly observed in PD, AD, and MCI cases [53,54,55]. The correlation between elevated Hcy concentrations and vitamins B_9_ and B_12_ deficiency might be explained by taking into account that both vitamins are involved in the methionine/homocysteine cycle [90]. Vitamin B_12_ is the cofactor of methionine synthase that catalyzes the methylation of Hcy leading to the formation of methionine and S-adenosylmethionine. Methionine synthase also uses 5-methyltetrahydrofolate as a one-carbon donor. In addition to that, folate-bound one-carbon units are necessary for purine synthesis mediated by deoxythymidine monophosphate. In this regard, an interesting study has shown that three-year folate supplementation improved cognitive functions such as memory, information processing speed, and sensorimotor speed, usually associated with dementia [91]. 

Serum Hcy concentration has been reduced by administrating vitamins B_6_, B_9_, and B_12_ in AD patients but no improvements in cognitive decline were observed [92,93]. In a case-control study, higher levels of vitamins B_9_ and B_12_ were shown to act as protective factors against AD [94]. On the other hand, no correlation between Hcy, folate, and vitamin B_12_ was observed in other studies [95,96]. The observed discrepancy may rely on different factors such as the sample size, duration, and control of the variables applied to the study.

In addition to the impact on Hcy concentration, the supplementation of vitamins B_6_, B_9_, and B_12_ reduced tau hyperphosphorylation and ameliorated the cognitive functions of adult mice exposed to hypobaric hypoxia [97]. In fact, vitamin B_12_ is able to interfere with several mechanisms associated with AD, such as amyloid precursor protein (APP) processing, amyloid β (Aβ) fibrillization, Aβ-induced oxidative damage, and tau hyperphosphorylation and tau aggregation [98]. Interestingly, the inhibition of tau fibrillization is mediated by the binding of vitamin B_12_ to Cys residues of the tau protein [99].

In addition to vitamin B_6_, B_12_, and folate, the role of riboflavin in slowing the progression of cognitive decline and reducing the risk of depression in aging is well accepted [100]. Intragastric riboflavin administration to double transgenic APP/PS1 mice, used as an in vivo AD model, led to a significant reduction in ROS by increasing SOD activity [101]. Riboflavin is also required for the flavin adenin dinucleotide (FAD)-dependent flavoenzyme methylenetetrahydrofolate reductase (MTHFR), involved in the one-carbon metabolism of Hcy remethylation. Impaired activity of MTHFR and its variant 677T leads to high Hcy plasma levels attributed to cellular FAD deficiency [102,103]. 

Thiamin deficiency was observed in AD cases by measuring plasma vitamin levels and the activity of the transketolase, a thiamin pyrophosphate-dependent enzyme [104,105]. The interest in thiamin is primarily related to numerous similarities occurring between classical thiamine deficiency and AD in terms of both cognitive deficits and reductions in brain glucose metabolism [106].

There are different outcomes in terms of niacin integration. Dietary niacin intake was shown to protect against AD and cognitive decline development [107]. On the other hand, the administration of reduced nicotinamide adenine dinucleotide (NADH), the biologically active form of niacin, showed no cognitive improvements in patients with different types of dementia, among which is AD [108]. The two studies primarily differ in the number of cases, the former being approximately 40 times greater. In 3xTg-AD mice, oral supplementation of niacinamide improved cognitive dysfunctions [109]. The observed ameliorations were caused by the reduction of Thr231 phosphorylated tau, which, in turn, increased tau degradation [109]. Thr231 phosphorylated tau is well known for its implication in AD and is also used as a biomarker for AD in cerebrospinal fluid (CSF) [110]. Moreover, the same study demonstrated that nicotinamide treatment affects acetyl-α-tubulin brain levels, thus promoting increased microtubule stability [109]. However, the positive effects exhibited by nicotinamide were completely reversed in late-stage mice with an existing severe pathology suggesting that nicotinamide treatment might be effective at the early or mild AD stages only. By using a different AD rat model, obtained by the administration of Aβ42 peptide into the rodents’ brains, intraperitoneal niacinamide caused the reduction of oxidative stress, apoptosis, and poly(ADP-ribose) polymerase-1 (PARP-1) activity, which are well known to be associated with neuroinflammation and cell death [111].

In addition to what has been previously reported for mammalian tauopathies, the brains of postmortem AD subjects manifested reduced levels of biotin carboxylase [38]. Intraperitoneal biotin and oral coenzyme Q_10_ (CoQ_10_) supplementation, both alone and in combination, attenuated neuroinflammation and improved brain insulin signaling in rats with AD induced by oral administration of AlCl_3_ [112].

Finally, the analysis of 18 human brains with short post-mortem delay revealed the reduction of vitamin B_5_ levels in different brain regions (hippocampus, entorhinal cortex, middle temporal gyrus, cingulate gyrus, sensory cortex, and cerebellum) [113]. The involvement of pantothenic acid in AD is further supported by the reduction in tricarboxylic acid cycle protein concentrations [114].

Compared to PD, the role played by vitamins C and E in AD is less controversial. Most of the studies indicate that vitamin C and E supplements play a protective role in the risk of AD [115] and that AD patients have lower serum, brain, and CSF levels of vitamins C and E than controls [95,116,117,118]. However, a prospective cohort study showed that upon administration of vitamin E and C, alone or in combination, the risk of dementia or AD was not decreased [119].

In a mouse model of AD, a 6-month treatment with vitamin C in a drinking solution attenuated Aβ oligomerization and behavioral deficits and reduced brain oxidative damage and hyper-phosphorylated tau proteins [120]. Oral supplementation of vitamin C (200 and 400 mg/kg body weight) protected against neuroinflammation-mediated neurodegeneration and memory deficits in a colchicine-induced rat model of AD. However, a higher dose (600 mg/kg body weight) worsened oxidative stress, neuroinflammation, and cognitive impairments [121]. On the other hand, an open clinical trial reported that supplementation with vitamin C and E had no beneficial effect in AD patients, except a limited antioxidant activity in the CSF [122] and, in a Japanese cross-sectional study, lymphocyte and plasma vitamin C levels were weakly correlated with the Mini-Mental State Examination Japanese version (MMSE-J) scores in AD individuals [123]. The different outcomes obtained for vitamin C might be explained by considering the prooxidant role exhibited by ascorbic acid, which can generate a high oxidative environment in the presence of redox-active metal ions, such as Fe and Cu [124].

In a randomized trial, the administration of α-tocopherol (2000 IU/d) for over two years slowed the functional decline in mild to moderate AD cases compared to a placebo group [125]. In a rat model of AD, intraperitoneal injection of vitamins E and D_3_, alone or combined, led to improved cognitive and memory impairments along with reduced neuronal loss and oxidative stress [126]. Vitamin E prevented increased ROS formation, protein oxidation, and neurotoxicity in Aβ42-treated neuronal cultures [127]. However, in a Mendelian randomization study by Liu et al., the authors reported no significant correlation between circulating vitamin E levels and AD risk in individuals of European descent [128]. In addition, vitamin E administration (a total population of 57 AD subjects) did not prevent oxidative stress and was found to be detrimental to cognitive activities in some cases [129].

Lower serum and plasma vitamin A levels were also found in AD patients compared to controls [130,131]. The treatment of cells with retinoic acid prevented Aβ production by inhibiting γ-secretase-mediated cleavage of APP via retinoic acid receptor-α and retinoid X receptor-α [132]. In a streptozotocin-induced AD mouse model, β-carotene administration in the form of a suspension was found to improve cognitive functions, inhibit acetylcholinesterase (AChE), and reduce Aβ-protein fragments [133].

Several studies demonstrated the association between lower serum levels of vitamin D and an increased risk of AD [134,135]. In a randomized, double-blind, placebo-controlled trial, 12-month administration of vitamin D_3_ was found to ameliorate cognitive activities and reduce Aβ-related biomarkers in AD older adults [136]. A 7-year follow-up study by Annweiler and colleagues revealed a correlation between a higher vitamin D dietary assumption and reduced risk of developing AD in older women [137]. In a mouse model of intracerebroventricular streptozotocin-induced sporadic AD, oral vitamin D_3_ administration improved cognitive activities, attenuated neuroinflammation and oxidative stress, and ameliorated cholinergic functions [138]. On the other hand, a randomized controlled trial by Stein and coworkers reported that high-dose vitamin D_2_ supplementation does not improve cognition or disability with respect to a low dose in mild-moderate AD patients [139].

Finally, a lower vitamin K_1_ daily intake was found in early-stage AD patients compared to controls [140]. In post-mortem human brains, higher menaquinone-4 (vitamin K_2_) levels were associated with a lower risk of AD [141]. Pretreatment with vitamin K_2_ solution had a protective role against Aβ42-induced neurotoxicity by activating autophagy and ameliorating mitochondrial function in a *Drosophila* model of AD [142]. In a model of Alzheimer’s cell damage, the pretreatment of PC12 cells with vitamin K_2_ led to a significant decrease in Aβ42, H_2_O_2_, ROS cytotoxicity, and cell apoptosis via the inactivation of the p38 MAP kinase pathway [143].

**Table 2:** Summary of the studies and the effects (↑ increase; ↓ decrease; ↔ no change) of vitamins in AD.

Vitamin	Type of Study	Intervention	Results	Ref.
B_6_, B_9_, B_12_	Clinical	Serum levels	↔ B_6_ ↓ B_9_, B_12_	[88]
B_9_, B_12_	Case-control	Plasma levels	↓ B_9_, B_12_	[89]
B_6_, B_9_, B_12_	RCT *	Supplementation	↑ B_6_, B_9_, B_12_ ↔ Cognitive func.	[37,92]
B_9_, B_12_	Cross-sectional	Serum levels	↑ B_9_, B_12_ ↓ AD risk	[94]
B_1_, B_2_, B_9_, B_12_, C, A	Cross-sectional	Plasma levels	↓ B_2_, C, A ↔ B_1_, B_9_, B_12_	[95]
B_6_, B_9_, B_12_	In vivo	Rodents	↑ B_6_, B_9_, B_12_ ↑ Cognitive func.	[97]
B_12_	In vitro	Tau	↑ B_12_ ↓ Fibrillization	[99]
B_2_	In vivo	Rodents	↑ B_2_ ↑ Cognitive func. ↓ Ox. stress	[101]
B_3_	Prosp. cohort	Dietary intake	↑ B_3_ ↑ Cognitive func.	[107]
B_3_	Clinical	Supplementation	↑ B_3_ ↔ Cognitive func.	[108]
B_3_	In vivo	Rodents	↑ B_3_ ↑ Cognitive func. ↑ Microtubule stability	[109]
B_3_	In vivo	Rodents	↑ B_3_ ↓ Ox. stress	[111]
B_7_	In vivo	*Drosophila*	↓ B_7_	[38]
		Rodents	↓ Biotin carboxylase	
	Ex vivo	Human AD brains	↓ Biotin carboxylase	
	In vitro	Neurons	↓ B_7_ ↑ Mitochondrial stress	
B_7_	In vivo	Rodents	↑ B_7_ ↓ Neuroinflammation	[112]
B_5_	Ex vivo	Human AD brains	↓ B_5_	[113]
C, E	Clinical	Supplementation	↑ C, E ↓ AD risk	[115]
E	Clinical	CSF, Plasma levels	↓ E	[117,118]
C, E	Prosp. cohort	Dietary intake	↑ C, E ↔ AD risk	[119]
C	In vivo	Rodents	↑ C ↑ Cognitive func. ↓ Ox. stress ↓ Aβ oligomerization	[120]
C, E	Clinical	Supplementation	↑ C, E ↔ AD	[122]
E	Clinical	Supplementation	↑ E ↓ Functional decline	[125]
E	In vitro	E18 neurons	↑ E ↓ Ox. stress	[127]
A, E	Case-control	Serum levels	↓ A, E	[130]
A	In vitro	Several cell lines	↑ A ↓ γ-secretase	[132]
A	In vivo	Rodents	↑ A ↓ AChE	[133]
	In silico	Molecular docking	A—A ChE	[134]
D	Rotterdam	Serum levels	↓ D ↑ AD risk	[135]
D_3_	Clinical	Supplementation	↑ D_3_ ↑ Neuroprotection	[136]
D	Clinical	Dietary intake	↑ D ↓ AD risk	[137]
D_3_	In vivo	Rodents	↑ D_3_ ↓ Ox.stress ↑ Cholinergic	[138]
K_2_	In vivo	*Drosophila*	↑ K_2_ ↑ Neuroprotection	[142]
K_2_	In vitro	PC12	↑ K_2_ ↓ Aβ42 cytotoxicity	[143]

* Randomized controlled trial.

## 3. Metals in Parkinson’s and Alzheimer’s Diseases

Several transition metal ions are known to play key roles in AD and PD [8]. Altered homeostasis of biometals such as zinc, copper, iron, and manganese is associated with high neurotoxicity and oxidative stress conditions typically observed in AD and PD cases [144,145,146,147,148,149,150].

**Figure 2:**
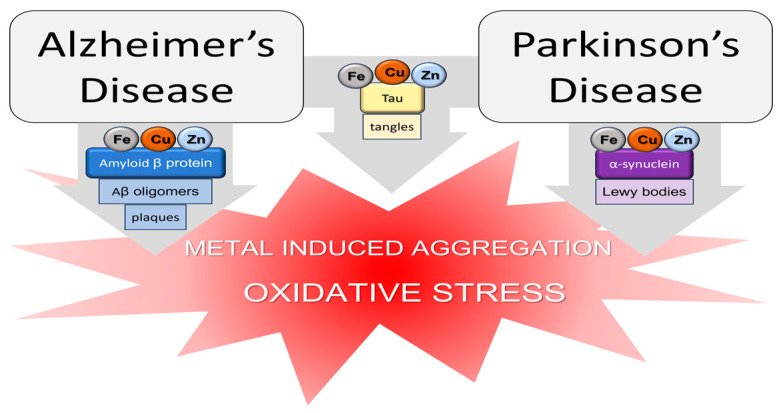
Schematic representation of the amyloidogenic proteins and their metal ion interactions in AD and PD.

In particular, redox-active metals such as Cu(II)/Cu(I) and Fe(III)/Fe(II) can catalyze the Fenton reaction, producing cytotoxic hydroxyl radicals from hydrogen peroxide [151,152]. In addition, copper and zinc are normally released at the glutamatergic synapse in the cortex and hippocampus and, together with iron, are able to bind amyloidogenic proteins and other hallmark molecules associated with NDs (Figure 2) [153,154,155,156]. Mn is a co-factor of glutamine synthetase involved in the recycling of glutamate to glutamine and thus responsible for the glutamate clearance from the synapse [157]. Mn is also essential for MnSOD activity protecting mitochondria from oxidative stress [157]. Other metal ions such as aluminum and nickel may represent risk factors for neurodegenerative diseases leading to mitochondrial dysfunction, microglial activation, and neuroinflammation [158,159]. Ni is extensively distributed in the environment. It is an essential nutrient for some animals, plants, and microorganisms, while its functional role in humans has not been recognized yet [160]. In contrast to Ni, Al is not an essential element. It is the most abundant metal on the earth’s crust and is widely used in daily human and industrial activities. Both Ni and Al traces can be found in food, drinking water, and the air.

As for vitamins, the interplay between metal ions and neurodegenerative diseases has been extensively investigated over the last thirty years. Research in this field has exponentially grown since 1990, reaching more than 3400 publications in the last ten years (Pubmed source “metal” and “neurodegeneration”). The scientific community has made great efforts to identify the role played by metal ions in the molecular associations and cellular pathways related to AD and PD. While much progress has been made in this area, several points remain to be clarified yet. In this review, we have focused on the relationship between these six metal ions and the two most common NDs, PD and AD, by briefly highlighting the metal’s coordination chemistry properties and metal involvement in AD and PD states.

### 3.1. Zinc, Copper, and Iron

Zinc, copper, and iron levels in serum, hair, CSF, and the brain have been extensively measured trying to correlate their content with metal dyshomeostasis associated with AD and PD cases [161]. The most applied techniques are atomic absorption, inductively coupled plasma atomic emission spectroscopy (ICP-AES), ICP-mass spectrometry (MS), and ICP-optical emission spectrometry (OES). Serum zinc levels were generally found to be reduced in patients affected by both AD and PD [162,163,164,165,166,167,168]. Decreased Zn concentrations have also been determined in AD hair samples [169]. On the other hand, reduced and increased copper contents have been measured in both AD [164,166,168,170,171] and PD cases [165,172]. Finally, a different behavior is displayed by iron, whose levels are different according to the disease, usually lower [166,167,173] or higher [165,174] in AD and PD patients, respectively.

Altered zinc, iron, and copper concentrations have also been found in CSF and post-mortem brains [161,175,176,177,178]. In PD patients, zinc levels are higher in the substantia nigra, caudate nucleus, and lateral putamen [175]. Iron content is higher in the substantia nigra and lower in the globus pallidus [175]. In PD, copper is increased in the putamen and decreased in the substantia nigra [175], while it is decreased in AD brains [178].

The altered metal levels observed in AD also correlate with the presence of Fe and Cu, Zn, in the AD senile plaques, primarily constituted by the aggregated forms of Aβ [179,180,181,182]. Aβ is a well-known amyloidogenic protein associated with AD and it is able to bind copper, zinc, and iron by means of His imidazole, N-terminal amino, and Glu/Asp carboxylate groups [183,184,185,186,187,188,189,190]. In a similar way, the amyloidogenic proteins tau and alpha synuclein, associated with AD and PD, respectively, can steadily coordinate several transition metal ions [191,192,193,194,195] (Figure 2).

Metal ions such as Cu and Zn can impact the aggregation of amyloidogenic proteins by affecting the morphologies and kinetics of the aggregates. The scientific community put a lot of effort into understanding the influence of metal ions, primarily copper and zinc, in the aggregation of amyloidogenic proteins [196,197,198,199,200,201,202]. The obtained findings are quite heterogeneous primarily due to the intrinsic complexity of the systems and different experimental conditions and techniques. In general, it is evident that zinc promotes the formation of amorphous Aβ aggregates while copper favors the production of highly cytotoxic oligomers [203,204,205]. As for Aβ, metal ion binding impacts the aggregation of αSyn as well, either showing pro- or anti-aggregatory effects [206,207]. Among all the metal ions, iron and copper are able to influence αSyn aggregation by promoting the formation of multimeric species and αSyn assembly [208,209].

Zinc interaction with the third repeat unit of the microtubule-binding domain of tau (R3tau) leads to the formation of Zn(II)-R3tau aggregates [210]. Such complexes, compared to R3tau, possess higher toxicity towards Neuro-2A (N2A) cells by inducing higher ROS generation in N2A cells. Copper increases the aggregation propensity of tau through its capability to both bind tau and produce ROS [211]. Zn and Fe binding to tau-R1 and R4 was also investigated; Zn(II) and Fe(II) but not Fe(III) coordination was demonstrated by CD and ESI-MS. Both interactions induced conformational changes in R1 and R4 [212]. Copper binding to tau occurs via His residues present in R1, R2, R3, and R4 or at the N-terminal site [213,214,215]. Recent molecular dynamic studies have revealed the misfolding of R3tau upon Cu(II) binding [216]. In addition, the ability of the copper–R3tau complex to promote the oxidation of dopamine has been recently reported [215].

The involvement of zinc, iron, and copper in AD is also supported by in vivo animal studies showing the effects of metal deficiency and/or supplementation in AD mice models [217,218,219,220,221]. For example, a zinc-deficient diet in an APP/PS1 mouse model of AD accelerated memory deficits through the induction of the NLRP3-inflammasome complex [217]. Other studies show that treatment with low levels of Cu(II) in drinking water led to an increase in Aβ production in neuroinflammation [218] and promoted Aβ accumulation, reducing mice’s cognitive functions [219]. Finally, in hypercholesteremia-induced AD rabbits, the administration of Fe(III) chelator deferiprone in drinking water significantly reduced the levels of plasma iron and cholesterol and decreased tau phosphorylation, Aβ40, and Aβ42 but not ROS production in the hippocampus [220]. In contrast, the treatment of an AD mouse model with Fe(II)-containing water markedly reduced Aβ42 deposition, tau phosphorylation, and apoptotic neurons and led to an increase in Aβ40 and a reduction in the Aβ42/Aβ40 ratio [222].

### 3.2. Manganese, Nickel, and Aluminum

The relevance of Mn, Ni, and Al in both AD and PD is well documented in the literature even if to a lesser extent than the essential Zn, Cu, and Fe ions. In PD, the serum levels of Mn, Ni, and Al are generally higher compared to healthy controls [163,174,223].

Furthermore, acute exposure to Mn can result in manganism, a type of parkinsonism, considered part of the PD etiology [221]. Manganism may be caused by elevated Mn accumulation in the basal ganglia region of the brain [224].

The association between aluminum and PD was suggested by the detection of Al in the Lewy bodies of PD patients, while its value is below the limit of detection in control brains [225]. Such findings are further supported by the higher incidence of ulcer patients that make high use of Al(III)-containing antiacids in PD cases compared to controls [226]. In addition, Al(III) was found to increase monoamine oxidase B and SOD activities in a way similar to what was observed in PD patients [163,227].

As for PD, higher serum levels of Ni [223] and Al [166,228,229,230,231] have been found for AD cases. On the other hand, reduced [167,232] or increased [169,233] Mn serum levels are reported for AD and MCI subjects. Mn content was also lower in the hair and nails of AD cases compared to control subjects [233]. Nickel levels were higher in the post-mortem frontal cortex and ventricular fluid of AD subjects with respect to nondemented elderly controls [234]. At the same time, nickel supplementation in the forms of the NiCl_2_ and NiCl_2_-morpholine complex prevented tau aggregation and promoted its degradation with the formation of shorter aggregates [235].

Moreover, in vitro and in vivo investigations on APP/PS1 mice showed dose-dependent neurotoxicity and an increase in Aβ upon Mn(II) treatment [236].

Finally, Al(III) may be implicated in AD pathogenesis via the induction of APP overexpression and the subsequent increase in Aβ and plaque formation in the brain [237]. A laser microprobe mass analysis showed a primary accumulation of Al(III) in the neurofibrillary tangles (NFTs) of AD subjects [238]. A 15-year follow-up study revealed an association between the high consumption of aluminum from drinking water and an increased risk of AD [239]. APP/PS1 transgenic mice, treated with intracerebroventricular microinjections of AlCl_3_, presented more extensive worsening of cognitive abilities and increases in neural apoptotic rates than APP/PS1 alone and wild-type mice exposed to Al [240].

## 4. Interaction between Vitamins and Metal Ions

**Figure 3:**
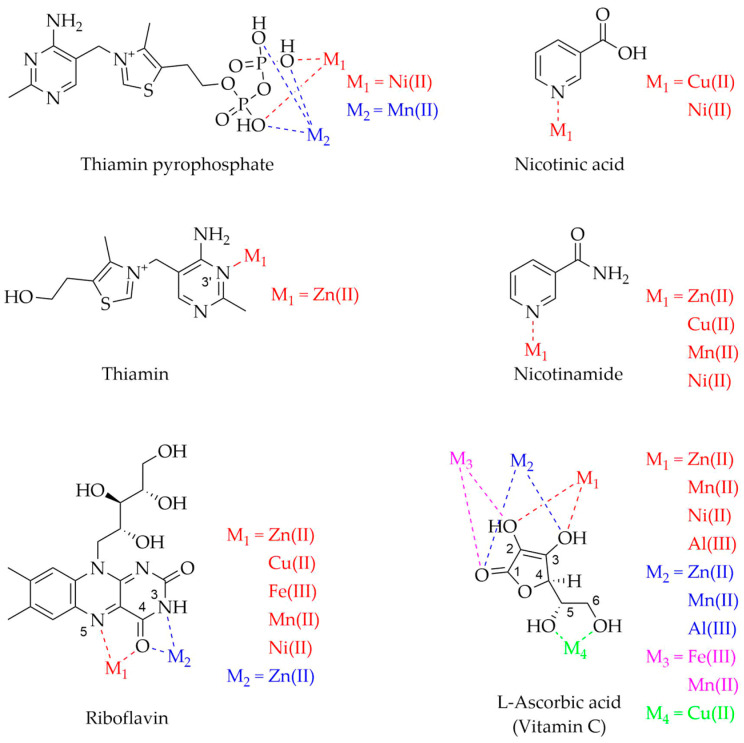
Metal complexes with thiamin, riboflavin, niacin and vitamin C. Thiamin pyrophosphate: M_1_ = Ni(II) [242,243], M_2_ = Mn(II) [244]. Thiamin: M_1_ = Zn(II) [245]. Riboflavin: M_1_ = Zn(II), Cu(II), Fe(III), Mn(II), Ni(II) [246], M_2_ = Zn(II) [247]. Nicotinic acid: M_1_ = Cu(II), Ni(II) [248]. Nicotinamide: M_1_ = Zn(II), Cu(II), Ni (II) [250], Mn(II) [250,251]. L-Ascorbic acid: M_1_ = Zn(II), Mn(II) [252], Ni(II) [253], Al(III) [253,254], M_2_ = Zn(II), Mn(II) [252], Al(III) [254], M_3_ = Fe(III), Mn(II) [255], M_4_ = Cu(II) [256].

**Figure 4:**
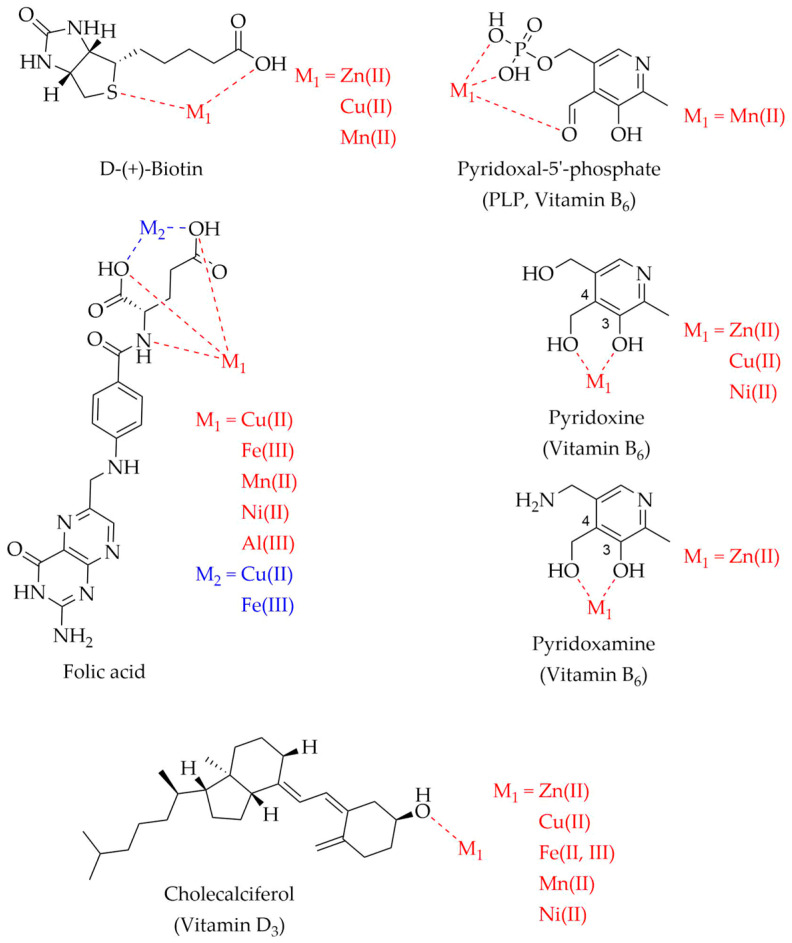
Metal complexes with biotin, folate, vitamins B_6_ and D_3_. D-(+)-Biotin. M_1_ = Zn(II), Cu(II), Mn(II) [262,263]. Folic acid: M_1_ = Cu(II), Fe(III), Mn(II), Ni(II), Al(III) [264], M_2_ = Cu(II), Fe(III) [265]. PLP: M_1_ = Mn(II) [257]. Pyridoxine: M_1_ = Zn(II) [260,261], Cu(II) [261], Ni(II) [258,261]. Pyridoxamine: M_1_ = Zn(II) [259]. Cholecalciferol: M_1_ = Zn(II), Fe(II, III), Mn(II) [264], Cu(II), Ni(II) [266].

**Figure 5:**
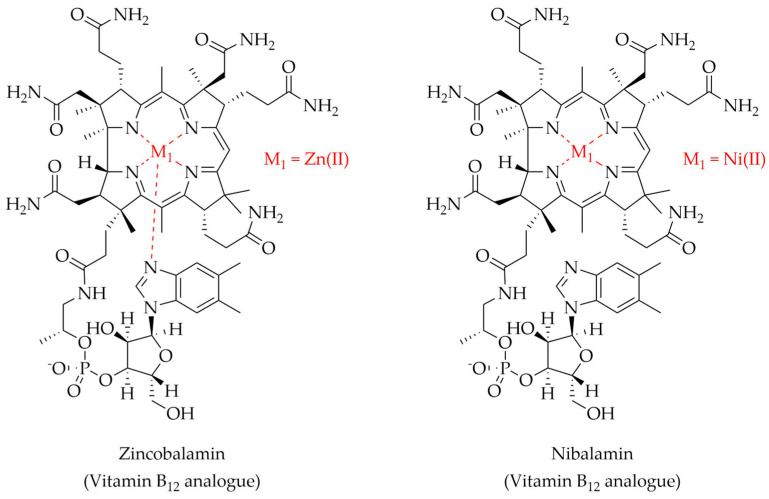
Metal complexes with vitamin B_12_. Zincobalamin: M_1_ = Zn(II) [267]. Nibalamin: M_1_ = Ni(II) [268].

After dealing with the implications of vitamins and metals in AD and PD, this paragraph will review the literature concerning the chemical interaction between vitamins and the selected metal ions, i.e., Zn(II), Cu(II), Fe(II), Fe(III), Mn(II), Ni(II), and Al(III), with the final aim to evaluate if vitamins might interfere with the metal–protein associations usually dysregulated in AD and PD (vide Section 3), thus possibly explaining the positive effects observed for various vitamins (vide Section 2). In fact, vitamins may interfere with the metal binding modes and the protein structural rearrangements as previously observed for other natural compounds [241]. Several papers addressed the synthesis, characterization, and evaluation of the thermodynamic and structural features of vitamin–metal complexes in vitro. Figure 3, Figure 4 and Figure 5 show the metal ions and identified vitamin donor atoms as described in detail hereafter. Table 3 lists all the metal–vitamin interactions studied so far together with the experimental techniques and conditions, such as to provide a solid starting base for researchers involved in developing this intriguing bioinorganic field. 

Research on the interaction between thiamin pyrophosphate and divalent metal cations reported different coordination modes by the ligand schematically represented in Figure 3. Ni(II) coordination occurs through the pyrophosphate group and a water molecule that bridges the metal to the ligand thanks to a hydrogen bond with N1′ [242,243]. On the other hand, Mn(II) is coordinated only with the pyrophosphate group, which is folded over the thiazolium ring [244].

The interaction between thiamin and Zn(II) was investigated by IR and NMR techniques revealing metal coordination at the N3′ position of the pyrimidine ring as shown in Figure 3 [245]. Indeed, a comparison of ^1^H NMR spectra of thiamin hydrochloride solutions with and without Zn(II) showed a significant upfield shift (0.28 ppm) and broadening of the amino group close to the binding nitrogen, while the ^13^C NMR revealed downfield chemical shifts of the carbons adjacent to the coordination site [245].

Extensive investigations on the riboflavin–metal ions interaction in an aqueous solution have indicated the ability of this vitamin to form 1:1 and 1:2 (ion:ligand) chelates with different metal cations, including Cu(II), Ni(II), Zn(II), Fe(III), and Mn(II) [246]. Metal coordination occurs via the carbonyl oxygen at the C4 position and N5 of the isoalloxazine moiety (Figure 3). A more recent study reported the synthesis and characterization of riboflavin metal complexes. In the case of Zn(II), the metal is bound through the oxygen at the C4 site and N3 of the ligand (Figure 3), with the addition of two water molecules to complete the metal coordination sphere [247].

The possibility for niacin to form complexes with divalent metal cations was investigated in several papers. Hernowo et al. studied the interaction of nicotinic acid with a number of metal ions, among them Cu(II) and Ni(II) [248]. Potentiometric measurements combined with software computations revealed higher stability for the copper complex, in line with the Irving–Williams series [249]. In addition, the authors predicted the molecular structure of both copper and nickel complexes in which metal coordination occurs via the pyridine nitrogen of nicotinic acid as shown in Figure 3 [248]. In a recent paper, nicotinamide was found to form coordination compounds with divalent metals such as Mn(II), Ni(II), Cu(II), and Zn(II) in a 2:1 ligand:metal ratio [250] (Figure 3). Furthermore, Sismanoglu reported the formation of 1:1 and 1:2 nicotinamide-Mn(II) complexes in which the pyridine N of the ligand binds Mn(II) ions [251].

Vitamin B_6_ has been widely investigated for its ability to coordinate metal ions. Pyridoxal-5′-phosphate was found to form a 1:1 (ligand:metal) complex with Mn(II), coordinating the metal via the phosphate group and the aldehyde oxygen as shown in Figure 4 [257]. Thermodynamic and kinetic parameters for the complexation reaction of pyridoxine with Ni(II) indicated a positive value of the enthalpy of interaction, suggesting an endothermic process for the formation of a pyridoxine-Ni complex [258]. Using X-ray analysis, Thompson and colleagues determined the structure of the Zn(II)-pyridoxamine complex [259]. The compound presents an octahedral geometry with the zinc atom chelated to the 4-aminomethyl and phenolate groups of two pyridoxamines (Figure 4). Two water molecules complete the octahedral structure. In addition, two nitrate groups in the secondary coordination sphere of zinc form hydrogen bonds with the water molecules and the 4-aminomethyl group of the ligand. Another metal complex involving vitamin B_6_ was reported for pyridoxine and Zn(II) ions [260]. Again, the geometry is octahedral with metal coordination occurring via the 4-hydroxymethyl and phenolate oxygens of two pyridoxines and two water molecules (Figure 4). On the other hand, Zhu and coworkers showed the formation of ML chelates, where M = Ni(II), Cu(II), or Zn(II) and L = pyridoxine, in which the metal is bound through the phenolic group at the C3 position and the hydroxyl at the C4 site (Figure 4) [261].

Studying the stability constants of binary 1:1 complexes of D(+)-biotin with Mn(II), Cu(II), and Zn(II) in various solvents, Sigel et al. determined the involvement of the carboxylic group in metal complexation [262]. In addition, for Mn(II) and Cu(II) complexes, NMR analysis revealed weak participation of the sulfur atom in the metal coordination, as suggested by line broadening of one of the two hydrogens of the methylene group next to the sulfur atom (Figure 4). Therefore, the authors proposed a stereospecific interaction between Mn(II) or Cu(II) and sulfur, where the metal binds the sulfur atom from below the plane [262]. Such a behavior of the thioether group was later confirmed in complexes involving D(+)-biotin and metal ions among which are Mn(II), Cu(II), and Zn(II). Again, metal coordination to sulfur was found to occur below the tetrahydrothiophene group, *trans* with respect to the imidazolidinone ring [263].

The complexing ability of folic acid with metal ions was largely studied by Yousef and coworkers. Potentiometric and conductometric studies showed the possibility of forming 1:1, 1:2, and 1:3 metal to ligand complexes for a series of metal ions, including Al(III), Fe(III), Cu(II), Mn(II), and Ni(II) [264]. The structure of the complexes is characterized by the presence of six-membered chelate rings. Folic acid binds the metal cation through the imino and the two carboxylic groups of the glutamic acid moiety (Figure 4). Other investigations pointed out the ability of folic acid to form two octahedral coordination compounds involving Cu(II) or Fe(III) as metal centers with a 1:2 metal-to-ligand ratio [265]. Folic acid was found to act as a bidentate ligand coordinating the metal ion through both carboxylic groups of the glutamate moiety and two water molecules in the coordination sphere (Figure 4). Polarimetric analysis revealed no angle of rotation for both complexes, suggesting a symmetric geometry.

NMR structural characterization of zincobalamin (Znbl), a Zn(II)-analogue of vitamin B_12_, revealed a structure similar to the Co(II) counterpart, i.e., Co(II)cobalamin (Cobl) (Figure 5) [267]. Using ^1^H-^1^H ROESY spectroscopy, the authors demonstrated that Znbl is isostructural to Cobl. In addition, the computationally generated structure of Znbl showed a downward movement of the 5,6-dimethylbenzimidazole moiety of the molecule with respect to Cobl. However, Znbl and Cobl were similar in overall architecture [267]. The cobalt ion in cobalamin was also replaced by Ni(II), generating nibalamin (Nibl). UV-Vis and NMR characterization suggested that Nibl is isoelectronically and roughly isostructurally analogous to Co(I)cobalamin [268].

Researchers extensively studied the interaction between vitamin C and metal ions. Tajmir-Riahi focused on the interaction of L-ascorbic acid with several metal ions both in a solution and in a solid state (Figure 3). Solid salts with the stoichiometry M(L-ascorbate)_2_^.^2H_2_O, where M = Zn(II) or Mn(II), and Al(L-ascorbate)_3_ were isolated and studied by ^13^C NMR and Fourier-transform infrared (FT-IR) spectroscopy techniques. The author demonstrated that, in an aqueous solution, Zn(II) and Mn(II) ions form chelates where the ascorbate binds the metal via its O2 and O3 atoms. In the solid state, two ascorbate anions were involved in the metal coordination through O2 and O3 of the first ion and O1 and O3 of the second one, together with two water molecules, leading to a six-coordinated complex [252]. In an aqueous solution, Al(III) was chelated by the O2 and O3 atoms of the ligand, while in the solid state, Al is bound via O1, O3, and O2, O3 of the anions, and water molecules [254]. In a two-part research study on the synthesis and characterization of metal-ascorbate complexes, Obaleye and coworkers reported paramagnetic behavior and an octahedral structure for coordination compounds involving vitamin C and Mn(II) and Fe(III) (Figure 3). Such complexes, with the formula Mn(L-ascorbate)_2_(H_2_O)_2_ and [Fe(L-ascorbate)_2_(H_2_O)_3_]Cl, showed a bidentate complexation mode of the ascorbate anion via its carbonyl and O2 atom [255]. Recently the complexation of Al(III) and Ni(II) by L-ascorbic acid was investigated using potentiometry, ^1^H NMR spectroscopy, and DFT calculations [253]. Experimental and theoretical data indicated that vitamin C preferentially coordinated the metal ions via the deprotonated oxygens at C2 and C3 positions (Figure 3). DFT computations suggested that Al(III) complexes were formed with an ion:ligand stoichiometric ratio of 1:2, while for Ni(II) compounds, the 1:1, 1:2, and 1:3 stoichiometries were possible. All the computed structures exhibited water molecules in the coordination sphere along with a prevalence of octahedral geometry. Ünaleroğlu and colleagues prepared a stable Cu(II)-ascorbate complex in CH_2_Cl_2_. The compound was found to be paramagnetic, and susceptibility measurements undoubtedly indicated a +2 oxidation state of the metal. ^1^H NMR and IR analyses revealed Cu coordination by the enolic and side chain oxygens of the ascorbate (Figure 3). One ligand unit binds the metal via the O2 and O3 atoms, and another one through the side chain oxygens, leading to a four-fold coordinated copper; such a structural unit repeats to form a polymer [256].

Mercê et al. carried out potentiometric studies of vitamin D_3_ complexes with various metal ions, including Mn(II), Fe(II), Fe(III), Zn(II), Ni(II), and Cu(II) in a water ethanol-medium [266,269]. The authors showed the formation of ML (M = metal, L = OH deprotonated vitamin D_3_) and ML_2_ species for all the cations reported above, except for Cu(II) (Figure 4). The absence of this complex was partially attributed to the formation of insoluble compounds at pH~5. For Cu(II) and Fe(III), potentiometric analysis allowed the detection of MLH species, where the ligand is protonated at the OH site. Moreover, Fe(III) was found to form an ML_3_ complex by deprotonating and holding a third ligand molecule.

**Table 3:** Summary of the interactions between vitamins and metal ions.

Vitamin	Metal Ions	Experimental Techniques	pH	References
Thiamin	Zn(II)	NMR, IR	-	[245]
	Mn(II)	NMR, EPR	6.6	[244]
	Ni(II)	NMR	6.9	[242,243]
Riboflavin	Zn(II)	PT	-	[246]
		FT-IR, LC-MS, AS	-	[247]
	Cu(II)	PT	-	[246]
	Fe(III)	PT	-	[246]
	Mn(II)	PT	-	[246]
	Ni(II)	PT	-	[246]
Niacin	Zn(II)	UV-Vis, FT-IR, TGA, CV, MSM, MPD	-	[250]
	Cu(II)	PT, SP, UV-Vis	5.0	[248]
		UV-Vis, FT-IR, TGA, CV, MSM, MPD	-	[250]
	Mn(II)	UV-Vis, FT-IR, TGA, CV, MSM, MPD	-	[250]
		PT, TPD	4.0	[251]
	Ni(II)	PT, SP, UV-Vis	5.0	[248]
		UV-Vis, FT-IR, TGA, CV, MSM, MPD	-	[250]
Vitamin B_6_	Zn(II)	X-ray	-	[259]
		X-ray, DTA, FT-IR	-	[260]
		PT, SP	2.4–7.4 ^a^	[261]
	Cu(II)	PT, SP	3.8–8.8 ^a^	[261]
	Mn(II)	NMR	6.2, 7.0	[257]
	Ni(II)	UV-Vis, MCV, TPD	-	[258]
		PT, SP	3.8–8.4 ^a^	[261]
Biotin	Zn(II)	PT, NMR	3.5–8.5 ^b^	[262]
		NMR, UV-Vis	2.0	[263]
	Cu(II)	PT, NMR	3.5–8.5 ^b^	[262]
		NMR, UV-Vis	2.0	[263]
	Mn(II)	PT, NMR	3.5–8.5 ^b^	[262]
		NMR, UV-Vis	2.0	[263]
Folate	Cu(II)	PT, conductometry	>4.0	[264]
		EA, AA, polarimetry, FT-IR, DAEB	7.6–7.8	[265]
	Fe(III)	PT, conductometry	>4.0	[264]
		EA, AA, polarimetry, FT-IR, DAEB	7.6–7.8	[265]
	Mn(II)	PT, conductometry	>4.0	[264]
	Ni(II)	PT, conductometry	>4.0	[264]
	Al(III)	PT, conductometry	>4.0	[264]
Vitamin B_12_	Zn(II)	NMR, UV-Vis, CD, F, MS, HPLC-DAD, X-ray, DFTC, SC	6.0	[267]
	Ni(II)	NMR, UV-Vis, CD, F, MS, HPLC-DAD, X-ray, DFTC, SC	6.0	[268]
Vitamin C	Zn(II)	NMR, FT-IR	6.0–7.0	[252]
	Cu(II)	NMR, MS, IR, TGA, EA, SDCu, MSM	-	[256]
	Fe(III)	EA, MMD, UV-Vis, IR, AS	8.0	[255]
	Mn(II)	NMR, FT-IR	6.0–7.0	[252]
		EA, MMD, UV-Vis, IR, AS	8.0	[255]
	Ni(II)	PT, NMR, DFTC	>4.0	[253]
	Al(III)	NMR, FT-IR	6.0–7.0	[254]
		PT, NMR, DFTC	>4.0	[253]
Vitamin D	Zn(II)	PT, SP	>7.0	[269]
	Cu(II)	PT, SP	>2.0	[266]
	Fe(II)	PT, SP	>7.0	[269]
	Fe(III)	PT, SP	>2.0	[269]
	Mn(II)	PT, SP	>8.5	[269]
	Ni(II)	PT, SP	>8.0	[266]

^a^ pH range for the formation constant determination. ^b^ pH for the calculation of the stability constants. Abbreviations: AA, atomic absorption; AS, antimicrobial screening; CD, circular dichroism; CV, cyclic voltammetry; DAEB, determination of absorption efficiency in the blood; DFTC, density functional theory calculations; DTA, differential thermal analysis; EA, elemental analysis; EIS, enzyme inhibition studies; EPR, electron paramagnetic resonance; F, fluorescence; FT-IR, Fourier-transform infrared spectroscopy; HPLC-DAD, high-performance liquid chromatography with diode-array detection; IR, infrared; LC-MS, liquid chromatography-mass spectrometry; MCV, method of continuous variation; MMD, magnetic moment determination; MPD, melting point determination; MSM, magnetic susceptibility measurements; NMR, nuclear magnetic resonance; PT, potentiometric titration; SC, software computations; SDCu, spectrophotometrical determination of Cu; TGA, thermogravimetric analysis; TPD, thermodynamic parameters’ determination; UV-Vis, ultraviolet–visible.

## 5. Conclusions

Both vitamins and transition biometals such as copper, iron, and zinc are essential for brain health and development [66,270,271]. They are involved in a plethora of molecular processes guaranteeing cellular functioning. At the same time, they are also regulated by sophisticated homeostatic machinery preventing the accumulation of toxic species inside the cell. 

For instance, copper is an essential metal ion for neural and glial function and it is necessary for the catalytic activity of antioxidant enzymes such as superoxide dismutase, and neurotransmitter-related enzymes, such as dopamine-β-monoxygenase [272]. Iron is implicated in oxygen delivery since it is an essential component of hemoglobin, and it is therefore crucial for brain metabolism. Zinc takes part in glutamatergic synaptic neurotransmission and is involved in the biological antioxidant system [273]. At the same time, copper and iron levels inside the cells need to be tightly controlled to avoid the overproduction of harmful ROS from copper/iron-catalyzed Haber–Weiss and Fenton reactions [274]. 

Water-soluble vitamins act as coenzymes capable of catalyzing a large number of chemical reactions essential for several cellular functions. For example, mitochondrial aerobic respiration and cellular energy production are dependent on the coenzymes derived from vitamins B_1_, B_2_, B_3_, and B_5_, which are directly involved in the citric acid cycle, the electron transport chain, and the resultant ATP formation. Indeed, vitamin B_5_ is a component of acetyl coenzyme A and is critical in the metabolism and synthesis of carbohydrates, proteins, and fats [271].

Whereas the brain is the most metabolically active organ in the body, B vitamins should have a particular impact on brain function and health. As for essential metal ions, their concentrations in the brain are tightly regulated by specific mechanisms ensuring vitamin transport across the blood–brain barrier and their correct distribution in the brain [275,276]. Moreover, as for copper, iron, and zinc ions, which are the three most abundant trace metals in the human brain [277], the vitamin concentration in the brain is usually higher than in the plasma [278].

The data reported in Table 3 have shown that all B vitamins share the ability to bind the selected metal ions, except vitamin B_5_. To our knowledge, no data have been collected so far on these associations; however, by considering the presence of carboxylic groups in their chemical structure, we would expect metal-vitamin B_5_ interactions comparable to the ones detected for vitamin B_9_. 

Recently it has been shown that vitamin B_9_ can bind the tau protein, reducing its aggregation and β-sheet structure content [279]. Docking analysis has shown that folic acid interacts with Ser235, Gln244, Thr245, His268, and Gln269 in the proximity of the R1 region [279]. Interestingly, this region is also able to coordinate Zn(II), Fe(II), and Cu(II) [212,215], whose anchoring site is located at His268. By considering that folic acid can coordinate Cu(II) as well, in the future, it might be interesting to evaluate vitamins’ influence on the Cu(II)–R1tau interaction.

The two coenzymes derived from vitamin B_2_, flavin mononucleotide (FMN) and FAD, are involved in several biological processes: (i) The synthesis, conversion, and recycling of other B vitamins (B_3_, B_6_, and B_9_) and (ii) the synthesis of all heme proteins, among which are those involved in electron transfer and oxygen transport and storage [271]. Interestingly, riboflavin can bind the metal ions involved in both PD and AD and are discussed herein (vide supra). The reported outcomes point out the occurrence of stable metal complexes, which might reduce vitamin B_2_ bioavailability leading to riboflavin deficiency in the disease state, which, in turn, would result in severe mitochondrial dysfunctions. Although such a hypothesis needs to be further explored, it is well supported by previous contributions on metal-induced alterations in vitamins in birds [280]. The interaction of vitamin C with iron and copper has been widely investigated; nowadays, it is well-accepted that these reactions are catalytic and have unit stoichiometry. In the presence of these two metal ions, ascorbic acid loses its antioxidant properties, favoring the production of ROS species [124]. Moreover, ascorbic acid is able to tightly associate with Aβ and binds to Aβ42 oligomers at sites in the D23-K28 region [281]. In this respect, further investigations on ternary systems (Aβ, Cu(II), and vitamin C) may be useful to explain the chemical equilibria and the species forming in the solution.

Among the fat-soluble vitamins, only vitamin D has shown the ability to bind metal ions such as Zn(II), Cu(II), Fe(III), Fe(II), Mn(II), and Ni(II). In addition to the well-known role of vitamin D in calcium and bone metabolism, its neuroprotective effects have been recently described. Moreover, vitamin D is able to modulate the biosynthesis of neurotransmitters and neurotrophic factors, and its receptors are widespread in brain tissue [282,283]. Curiously, a recent study has investigated physical and chemical factors affecting vitamin D degradation, among which are metal ions such as Fe(II), Cu(I), and Cu(II) [284]. The obtained findings point out the occurrence of quick metal ion-mediated vitamin D_3_ degradation, regardless of the particular metal ion and its concentration. Metal-dependent degradation of vitamin D might be associated with the low levels of vitamin D found in both PD and AD (Figure 1).

In conclusion, this review points out the puzzling and fascinating role played by vitamins and metal ions in AD and PD. In this scenario, a new field focused on the bioinorganic chemistry of vitamins is emerging with the opportunity to illustrate the contribution of vitamin–biometal interactions to the well-known pathological implications associated with these two severe diseases.

## Data Availability

No new data were created or analyzed in this study. Data sharing is not applicable to this article.
